# Stress Response Simulated by Continuous Injection of ACTH Attenuates Lipopolysaccharide-Induced Inflammation in Porcine Adrenal Gland

**DOI:** 10.3389/fvets.2020.00315

**Published:** 2020-06-26

**Authors:** Zhiyuan Sun, Demin Cai, Xiaojing Yang, Yueli Shang, Xian Li, Yimin Jia, Chao Yin, Huafeng Zou, Yunming Xu, Qinwei Sun, Xuhui Zhang

**Affiliations:** ^1^Department of Animal Husbandry and Veterinary Medicine, Jiangsu Vocational College of Agriculture and Forestry, Jurong, China; ^2^Co-innovation Center for Sustainable Forestry in Southern China, College of Forestry, Nanjing Forestry University, Nanjing, China; ^3^Department of Biochemistry and Molecular Medicine, School of Medicine, University of California, Davis, Sacramento, CA, United States; ^4^College of Animal Science and Technology, Yangzhou University, Yangzhou, China; ^5^Key Laboratory of Animal Physiology and Biochemistry, Ministry of Agriculture, Nanjing Agricultural University, Nanjing, China; ^6^Laboratory of Animal Clinical Pathophysiology, Department of Animal Science and Technology, Shanghai Vocational College of Agriculture and Forestry, Shanghai, China; ^7^College of Animal Science and Technology, Jiangxi Agricultural University, Nanchang, China

**Keywords:** stress, toll-like receptor, glucocorticoid, lipopolysaccharide, adrenocorticotropine

## Abstract

On modern farms, animals are at high risk of bacterial invasion due to environmental stress factors. The adrenal gland is the terminal organ of the stress response. The crosstalk between adrenal endocrine stress and innate immune response is critical for the maintenance of immune homeostasis during inflammation. Thus, it's important to explore whether stresses play a pivotal role in lipopolysaccharide (LPS)-induced inflammatory response in the porcine adrenal gland. Thirty-days-old Duroc × Landrace × Large White crossbred piglets (12 ± 0.5 kg) were randomly allocated into four groups in a 2 × 2 factorial arrangement of treatments, including ACTH pretreatment (with or without ACTH injection) and LPS challenge (with or without LPS injection). Each group consisted of six male piglets. The results showed that our LPS preparation alone induced mRNA expressions of IL-1β, IL-6, TNF-α, IL-10, COX-2, TLR2, TLR4, and GR (*P* < 0.05). ACTH pretreatment downregulated the TLR2 mRNA and IL-6 protein level induced by our LPS preparation significantly (*P* < 0.05) by one-way ANOVA analysis. Treatment with LPS alone extremely significantly decreased ssc-miR-338 levels (*P* < 0.01). Interaction of ACTH × LPS was significant for cNOS level (*P* = 0.011) and ssc-miR-338 expression (*P* = 0.04) by two-way ANOVA analysis. The LPS treatment significantly downregulated cNOS levels (*P* < 0.01), which was significantly attenuated by ACTH pretreatment (*P* < 0.05). Lipopolysaccharide alone did not affect ssc-miR-146b expression levels compared to that in the vehicle group. However, ACTH pretreatment in combination with LPS significantly increased this micro-RNA expression (*P* < 0.05). TLRs 1–10 were all expressed in adrenal tissue. The LPS challenge alone induced remarkable compensatory mitochondrial damages at the ultrastructural level, which was alleviated by ACTH pretreatment. Accordingly, ACTH pretreatment was able to block LPS-induced secretion of local adrenal cortisol (*P* < 0.05). Taken together, our results demonstrate that ACTH pretreatment seems to attenuate LPS-induced mitochondria damage and inflammation that decreased cNOS activity in the adrenal gland and ultimately returned local adrenal cortisol to basal levels at 6 h post LPS injection.

## Introduction

Issues existing in modern farm transportation and management can induce various stresses in domestic animals (1, 2), which are associated with increasing incidence of diseases (3). Effects of stress on the immune response, reported in previous studies, are conflicting. For instance, high-yield dairy cows in the transition period suffered from metabolic stress, characterized by occurrence of an inflammatory response (4). Social disruption and an acute stressor have been shown to activate an inflammatory response in mice (5, 6). Lipopolysaccharide (LPS) challenge was shown to reduce feed intake and increase plasma pro-inflammatory cytokines of pigs, which was inhibited by high-temperature stress (7). Long-term effects of social stress have been reported to inhibit antiviral immunity in pigs (8). Generally, exposure to intense acute or long-term chronic stress may compromise host immune responses (9).

The adrenal gland is the terminal organ of the stress response, which can directly respond to acute and chronic stress (10). The adrenal response to stress is crucial for the host defense against infection (11). The initial step of host defense against bacterial infections is through pattern-recognition receptors, such as toll-like receptors (TLRs). In fact, the stress response and the innate immune response is coordinated by TLRs in the adrenal gland, which is crucial for animal survival during severe inflammation (11, 12). The LPS treatment induced expression of TLR2 and TLR4 in the adrenal gland of animals (12, 13). Most TLRs are responsible for the recognition of a variety of pathogens and induce inflammatory responses. During inflammation, release of cytokines is accompanied by a high glucocorticoid (GC) output. The inflammation is normally restricted by GC as a feedback mechanism (14). The GC acts through a ligand-dependent transcription factor glucocorticoid receptor (GR) (14, 15). Previous studies on mice have shown that deletion of TLR2 or TLR4 is associated with marked cellular alterations in adrenocortical tissue and an impaired adrenal corticosterone response (11, 12). Stress can stimulate the adrenal gland to release GC (16). However, little information is available regarding effects of stresses on LPS-induced inflammatory response via TLRs and GR in the adrenal gland of pigs.

The miRNAs act as a class of endogenous non-coding RNA. They can regulate inflammation via inhibiting mRNA transcription or promoting mRNA degradation of the target gene. It was reported that miR-338 was involved in regulating inflammation (17, 18). Previous studies have shown that some miRNA families, such as let-7 and miR-146, can directly decrease TLR4 expression and inhibit inflammation and oxidative stress response (19–22). In view of the importance of the adrenal response, here we aimed to investigate, for the first time, the effect of stress on LPS-induced inflammatory response in the pig adrenal gland. Usually continuous ACTH treatment is used to mimic the stress response (23, 24). Therefore, we used a pig model exposed to LPS injection with or without continuous ACTH pretreatment to study secretions of inflammatory cytokines, enzymes regulating oxidative stress, and cortisol as well as levels of expression of TLR2, 4, GR, and all miRNAs that target TLR2 and TLR4.

## Methods

### Animals and Experimental Design

This study was conducted according to “Guidelines on Ethical Treatment of Experimental Animals” (2006) No. 398 set by the Ministry of Science and Technology, China, and the “Regulation Regarding the Management and Treatment of Experimental Animals” (2008) No. 45 set by the Jiangsu Provincial People's Government. All animal procedures were approved by the Institutional Animal Care and Use Committee (IACUC) of Nanjing Agricultural University. A 2 × 2 factorial design was used (i.e., LPS treatment as one factor and ACTH treatment as the other factor). Twenty-four 30-days-old *Duroc* × *Landrace* × *Large White* crossbred piglets with an average weight of 12 ± 0.5 kg were obtained from a commercial farm. The herd in the farm had been monitored regularly during the last 5 years for infectious diseases in the sows and pigs of different age groups prior to our treatment, and no clinical and pathological evidence was found. Before the experiment, all the piglets were acclimatized for a week. All the piglets were randomly allocated into four groups in a 2 × 2 factorial arrangement of treatments, including ACTH (Sigma-Aldrich, Dublin, Ireland) pretreatment (with or without ACTH injection) and LPS challenge (with or without LPS injection): (1) vehicle group (ACTH- LPS-), (2) LPS injection without ACTH pretreatment group (ACTH- LPS+), (3) ACTH alone treatment group (ACTH+ LPS-), and (4) LPS injection with ACTH pretreatment group (ACTH+ LPS+). Each group consisted of six male piglets. The pigs were randomly divided into pens in four separate rooms equipped with appropriate air filters, and the vehicle group was retained in a pen distant from the other groups. The pens had fully slatted floors with natural light conditions. All pigs were provided with water and food *ad libitum*. They were fed a commercial feed with no antimicrobials throughout the trial, consisting of 65% corn, 24% soybean meal, 5% bran, 2% fish meal, and 4% premix, and the nutrition content included 18% crude protein and 3.4 Mcal/kg digestion energy in the diet formula, respectively. Because continuous ACTH administration to the animal was showed to be similar to stress in some studies (23, 24), all the ACTH pretreatment pigs were injected intramuscularly with ACTH (2.25 IU/kg body weight) for seven consecutive injections at 6-h intervals. The first injection started at 9 a.m. on the 1st day. As a classic ligand for TLR4, LPS stimulates the body to produce an inflammatory response (25, 26). The LPS from Escherichiacoli serotype K-235 (phenolextracted) (Sigma-Aldrich) was dissolved in 0.9% NaCl solution (27). Then all the LPS treatment pigs were injected intramuscularly with LPS (15 μg/kg weight) at 9 a.m. on the 3rd day post the first ACTH treatment. Pigs in the other two groups were mock injected with saline in the same manner. All pigs were euthanized at 6 h post-LPS treatment. The pigs were slaughtered by a head-only electric stun tong apparatus, followed by manual exsanguination. Microscopy histological analysis and levels of cortisol, cytokine protein, gene mRNA expression, and miRNA expression in the adrenal gland tissue were determined by the same person who was blinded to the experiment design.

### Sample Collection

These key sampling times post LPS injection were based on previous research in our laboratory (27, 28). All blood samples were collected from the precaval vein at 6 h post LPS treatment. Blood samples were centrifuged at 1,500 × *g* for 15 min at 4°C, and the serum was stored at −20°C. The adrenal gland tissue was harvested immediately after euthanasia. Subsequently, tissues were washed with PBS to remove any blood and contaminants on their surface. The samples were snap frozen in liquid nitrogen and stored at −70°C.

### Electron Microscopy

Adrenal glands were diced into small pieces at approximately 1 mm^3^ and fixed in 0.1 M phosphate buffer at pH 7.2–7.4 with 2% (vol/vol) glutaraldehyde. After washing in 0.2 M phosphate buffer (pH 7.2) overnight, these specimens were post-fixed in cold 1% OsO4 (pH 7.3) for 2 h at 4°C. All tissue slices were dehydrated in different gradients of ethyl alcohols (30–100%) and then embedded in Epon 812 R (Merck, Whitehouse Station, NJ). Finally, ultrathin sections (50 nm) were stained with uranyl acetate and lead citrate and examined by an H-7650 transmission electron microscope (Hitachi High-Technologies Co., Japan) at 80 kV with an Ultrascan CCD camera (11). Ultrastructure morphometric assessments of mitochondria were conducted by using NIH ImageJ software.

### Measurement of Markers for Oxidative Stress

All adrenal gland tissues were homogenized in cold radioimmunoprecipitation assay (RIPA) buffer of (50 mM Tris–HCl pH 7.4, containing 10% glycerol, 1.0% Triton-X 100, 100 mM NaCl, 50 mM NaF, 1 mM EDTA, and 1 mM EGTA) with the protease inhibitor cocktail (Roche Applied Science) then centrifuged at 1,500 × g for 15 min at 4°C, and the supernatant was extracted. The enzyme activities of total superoxide dismutase (T-SOD), catalase (CAT), inducible nitric oxide synthase (iNOS), constitutive nitric oxide synthase (cNOS), total nitric oxide synthase (TNOS), and XOD (xanthine oxidase) content in the supernatant of adrenal tissues was determined using biochemical determination kits (Nanjing Jiancheng Bioengineering Institute, Nanjing, China) (29–31).

### Cortisol and Cytokine Protein Levels in Adrenal Gland Tissue

Concentrations of cortisol and IL-6 in the supernatant of adrenal gland tissues at 6 h after LPS injection were measured in duplicate using a commercial 125I-RIA kit (Beijing Research Institute of Biotechnology, Beijing, China) according to the manufacturer's instructions. This kit was validated for measuring porcine samples (15, 31). The detection limits of cortisol and IL-6 were 2 ng/mL and 50 pg/mL, respectively. All samples were measured in the same assay to avoid inter-assay variations.

### Gene Expression Levels in Adrenal Gland Tissue

Briefly, total RNA was extracted from adrenal gland tissues using Trizol reagent (Invitrogen, Carlsbad, CA) according to our previous description (15). From each sample, 1 μg of total RNA was converted to cDNA using the PrimeScript® RT reagent kit with gDNA Eraser (Takara, Dalian, China). The primers used are listed in Table 1. Also, real-time quantitative PCR (QPCR) reactions and gene expression levels of mRNA were performed using the SYBR Green QPCR Master Mix (TOYOBO Ltd., Japan) as previously described (15). Glyceraldehyde-3-phosphate dehydrogenase (GAPDH) was chosen as a reference gene for normalization. All the mRNA expression levels in the adrenal gland tissues at 6 h post LPS injection were presented as the fold change relative to the average values in the Vehicle group (Supplementary Material S1).

**Table 1:** Nucleotide sequences of specific primers used in qPCR.

**Genes**	**Primers**
TLR1	5′–GTGTTGCCAATCGCTCAT−3′ 5′–CAGATTTACTGCGGTGCT−3′
TLR2	5′–GACACCGCCATCCTCATTCT−3′ 5′–CTTCCCGCTGCGTCTCAT−3′
TLR3	5′–TGCACTAAAACGTGAAGAACTT−3′ 5′–ATGAAAACACCCTGGAGAGAAC−3′
TLR4	5′–TCTACATCAAGTGCCCCTAC−3′ 5′–TAAATTCTCCCAAAACCAAC−3′
TLR5	5′–AGATACCCCTTGTGTGCGA−3′ 5′–TTCCTTGTGGTGTCCGCTG−3′
TLR6	5′–AGAAAGAAATCTTGAATTTGGA−3′ 5′–AATGAAGGCTTATGACAGTAGG−3′
TLR7	5′–TATGGGACCAGGAGCACACAA−3′ 5′–AAAGAGAACTGCCGATAGGGA−3′
TLR8	5′–CGGTCGCTTCCCACATC−3′ 5′–CCAGTCCCTCTCCTCCAAAC−3′
TLR9	5′–GGATGTGGGCTGAGGGAG−3′ 5′–AGGCTTTTGGGGAGGTTG−3′
TLR10	5′–TGTGGTATTGTCATGTCAGTGC −3′ 5′–AGTTGAAAAAGGAGGTTGTAGG−3′
GAPDH	5′–CGTCCCTGAGACACGATGGT−3′ 5′–CCCGATGCGGCCAAAT−3′
GR	5′–TCTGTATGAAAACCTTACTGCT−3′ 5′–TGTTCTTATCCAAAAATGTCTG−3′
IL-1β	5′–CAGGGGACTTGAAGAGAG−3′ 5′– GCTGATGTACCAGTTGGG−3′
IL-6	5′–CTACTGCCTTCCCTACCC−3′ 5′–ACCTCCTTGCTGTTTTCA−3′
TNF-α	5′–CCTCTTCTCCTTCCTCCT−3′ 5′–ATTGGCATACCCACTCTG−3′
IL-10	5′–CATCCACTTCCCAACCAG−3′ 5′–TCCTCCCCATCACTCTCT−3′
COX-2	5′–GTGTGAAAGGGAGGAAAGA−3′ 5′–AAACTGATGGGTGAAGTGC−3′
Ssc-miR-338	TCCAGCATCAGTGATTTTGTTG
Ssc-miR-146b	TGAGAACTGAATTCCATAGGC
Oligo dT-adaptor	TAGAGTGAGTGTAGCGAGCACAGAATTAATA CGACTCACTATAGGTTTTTTTTTTTTTTTTVN
Exogenous reference	GTGACCCACGATGTGTATTCGC
Universal	TAGAGTGAGTGTAGCGAGCA

### miRNA Expression Level in Adrenal Gland Tissue

All miRNAs that target TLR2 and TLR4 were predicted by computer-aided algorithms from TargetScan (http://www.targetscan.org/vert_42/), and the miRNA expression level was performed according to previous publication (32). Briefly, total RNA was extracted from the adrenal gland tissues using Trizol reagent. The RNA (4 μg) was polyadenylated by poly(A) polymerase (PAP) at 37°C for 1 h in a 20-μl reaction mixture using the Poly(A) Tailing Kit (AM1350, Ambion, USA), and tailing reactions were performed. The tailing reaction solution contained 4 μg of RNA samples (1 μg/μl), 2 μl of 25 mM MnCl2, 4 μl of 5 × E-PAP buffer, 0.8 μl of E-PAP, 2 μl of 10 mM ATP, and 7.2 μL of nuclease-free water in 20 μl final volume. Then, tailing RNAs (2 μg) were converted to cDNA using a gene-specific oligo dT-adapter primer (1 μg/μl). QPCR reactions were performed using the SYBR Green QPCR Master Mix (TaKaRa, Tokyo, Japan) with an Mx3000P QPCR system (Agilent Technologies, Stratagene, USA). All special miRNA primers were designed based on mature miRNA sequences of pigs in miRbase (http://www.mirbase.org/). All primers used are listed in Table 2, including special miRNA primers, universal primers, exogenous reference primers, and oligo dT-adaptor primers. All the miRNA expression levels in the adrenal gland tissues at 6 h post LPS injection were presented as the fold change relative to the average values in the vehicle group.

**Table 2:** Effects of LPS/ACTH on oxidative stress response in local porcine adrenal tissue.

**Items**	**ACTH -**		**ACTH** **+**		**S.E.M**.	***P*****–value**		
	**LPS -**	**LPS+**	**LPS -**	**LPS+**		**ACTH**	**LPS**	**ACTH × LPS**
T-NOS (U/mg protein)	1.70^a^	1.12^b^	1.38^ab^	1.42^ab^	0.08	0.955	0.089	0.052
iNOS (U/mg protein)	0.36	0.43	0.31	0.47	0.02	0.922	0.048	0.422
cNOS (U/mg protein)	1.34^a^	0.69^c^	1.07^ab^	0.95^b^	0.07	0.517	<0.010	0.011
T-SOD (U/mg protein)	87.94^a^	57.94^b^	76.28^a^	55.08^b^	2.59	0.215	<0.010	0.446
XOD (U/g protein)	35.58	28.63	32.91	32.02	1.77	0.901	0.187	0.304
CAT(U/mg protein)	0.90	0.88	0.80	0.90	0.03	0.515	0.55	0.328

*Means with different letters (a, b, c) are significantly different (P <0.05) from each other, n = 6. Different superscript letters indicate significant differences between the same column. Data are expressed as mean ± SEM*.

### Statistical Analysis

All data were analyzed in the general linear model (GLM) procedure of SPSS 16.0 (SPSS Inc., Chicago, IL, USA). Descriptive statistics were performed to check the normality and homogeneity of variances before using parametric analyses. IL-6 mRNA, TNF-α mRNA, IL-10 mRNA, IL-6 protein, and cNOS secretion levels were not normally distributed. Therefore, Log10 transformation was performed for these results before statistical analysis. All data were analyzed by two-way ANOVA in the general linear model (GLM) procedure of SPSS 16.0 (SPSS Inc.) with main effects of LPS and ACTH treatments and interaction of LPS × ACTH. For all data, a one-way ANOVA was performed also. Duncan's test was used as the *post-hoc* test. Two-tailed *P*-values with *P* ≤ 0.05 were considered significant. Data are expressed as mean ± *SEM*.

## Results

### Clinical Symptoms and Ultrastructure Change of the Adrenal Gland

**Figure 1:**
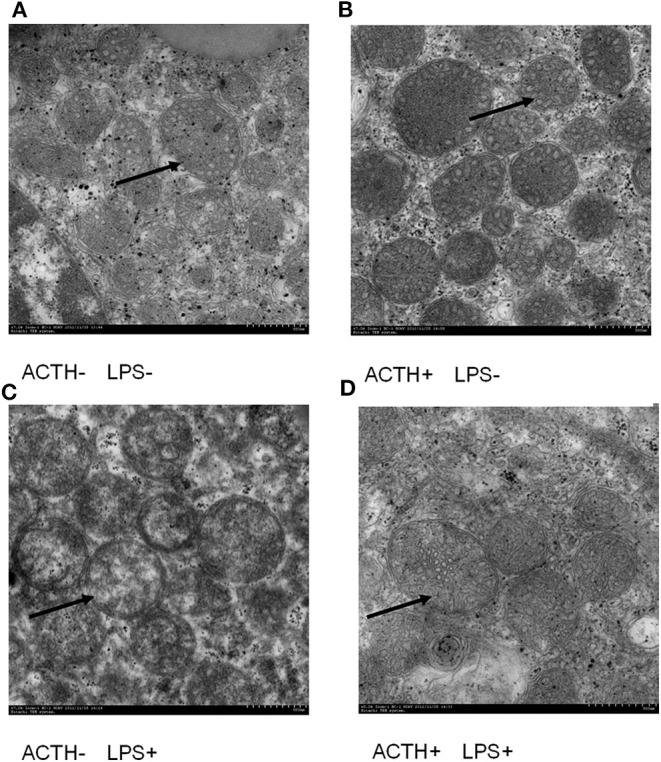
Effect of lipopolysaccharide/ACTH treatment on ultrastructure change of pig adrenocortical cells (arrow, scale bars = 500 nm). **(A)** Vehicle group and **(B)** ACTH groups show rod-like or round mitochondrias with characteristic tubovesicular cristae (arrow). **(C)** LPS group shows extremely round mitochondrias with a smooth outer membrane and disappeared cristae (arrow). **(D)** ACTH + LPS group shows mitochondrial cristae with tubovesicular structure (arrow).

LPS injection alone induced severe clinical symptoms in ACTH- LPS+ pigs. Five pigs lied down on the ground with signs of depression not drinking water within 6 h post LPS injection, and one pig was dying in the group although pigs in the ACTH+ LPS+ group showed mild clinical symptoms post ACTH pretreatment. Two pigs in the combined treatment group lied down on the ground with depressed spirits within 3 h post LPS injection but recovered quickly. The other four pigs moved and drank normally in the ACTH+ LPS+ group. All pigs in the other groups without LPS treatment did not show any clinical symptoms. At the ultrastructural level, the most pronounced effect was found in the mitochondrial architecture. All the animals without LPS treatment exhibited a rod-like or round mitochondria with characteristic tubovesicular cristae. In contrast, LPS treatment alone induced compensatory damage of the mitochondrial substructure, and mitochondrias became swollen and extremely round with a smooth outer membrane and disappeared cristae. The ACTH pretreatment obviously alleviated LPS-induced mitochondria injury and restored mitochondrial cristae to the tubovesicular structure (Figure 1).

### Profiles of Various Cytokines and Cortisol

**Figure 2:**
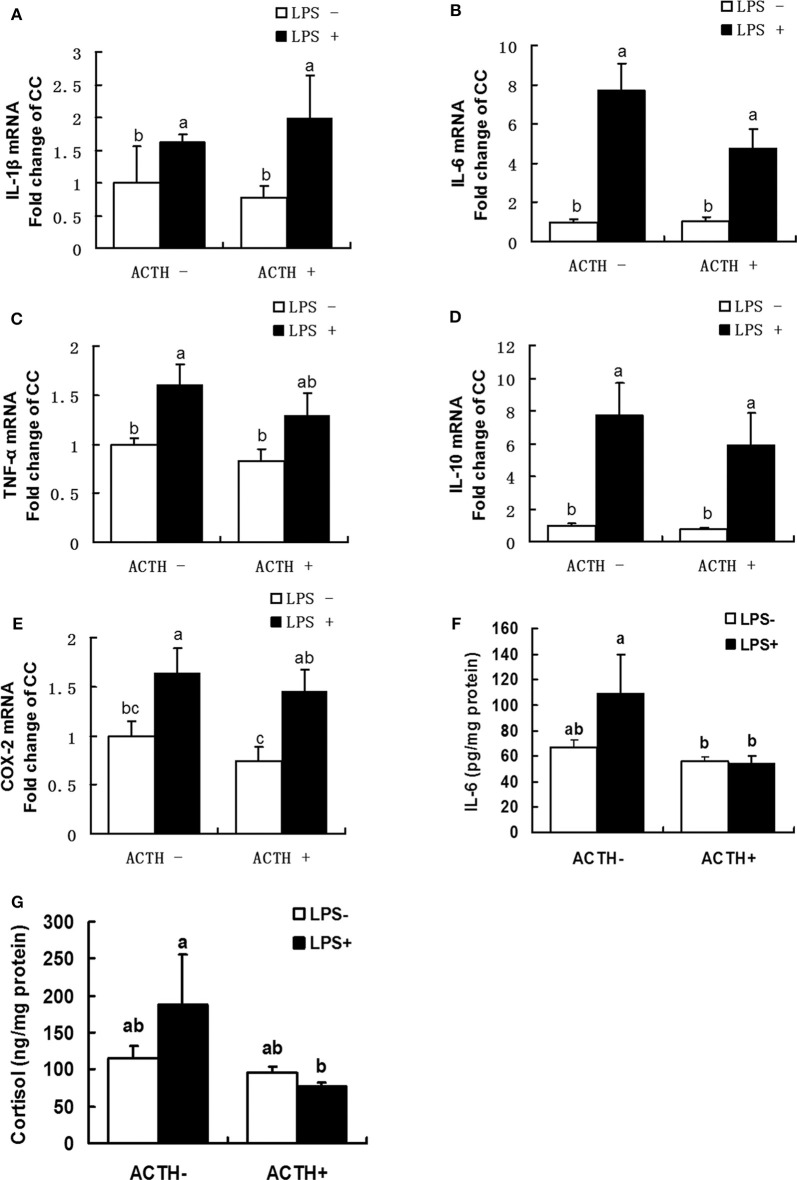
Effects of lipopolysaccharide/ACTH on expressions of IL-1β **(A)**, IL-6 **(B)**, TNF-α **(C)**, IL-10 **(D)**, and COX-2 **(E)** mRNAs, IL-6 protein **(F)**, and cortisol **(G)** secretion levels in local porcine adrenal gland tissue. Means with different letters (a, b, c, d, e, f) are significantly different (*P* < 0.05) from each other, *n* = 5–6. Different superscript letters indicate significant differences between the same column. Data are expressed as mean ± *SEM*.

As shown in Figure 2, a strong main effect of LPS was detected (*P* < 0.01). Treatment with LPS alone upregulated mRNA expressions of IL-1β, IL-6, TNF-α, IL-10, COX-2 (*P* < 0.05). Furthermore, ACTH pretreatment significantly inhibited LPS-induced IL-6 protein secretion (Figure 2F, *P* < 0.05). Consistent with this, ACTH pretreatment significantly alleviated the release of cortisol induced by LPS (Figure 2G, *P* < 0.05) and restored local adrenal cortisol to the basal level (Supplementary Table S1).

### Oxidative Stress in the Adrenal Gland

As shown in Table 2, LPS treatment revealed a main effect on T-SOD activity (*P* < 0.01). The LPS treatment significantly decreased T-SOD activity (*P* < 0.05); however, ACTH pretreatment did not restore T-SOD activity. Treatment with LPS increased levels of iNOS protein secretion in the adrenal gland (*P* = 0.048). There were a main effect of LPS (*P* < 0.01) and a ACTH × LPS effect (*P* = 0.011) for cNOS levels by two-way ANOVA. The LPS treatment significantly downregulated cNOS level (*P* < 0.01), which was significantly attenuated by ACTH pretreatment (*P* < 0.05) (Supplementary Table S1).

### Levels of Toll-Like Receptors and Glucocorticoid Receptor mRNA Expressions

**Figure 3:**
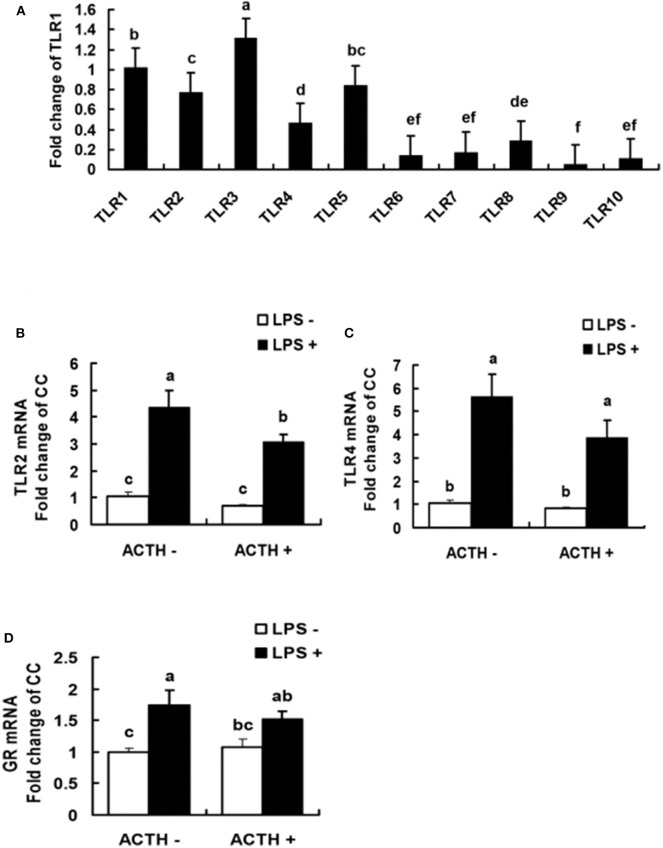
Levels of TLR mRNA in normal porcine adrenal tissue **(A)**, and effects of lipopolysaccharide/ACTH on mRNA expressions of TLR2 **(B)**, TLR4 **(C)**, and GR **(D)** in local porcine adrenal gland tissue. Means with different letters (a, b, c, d, e, f) are significantly different (*P* < 0.05) from each other, *n* = 5–6. Different superscript letters indicate significant differences between the same column. Data are expressed as mean ± *SEM*.

All the 10 TLRs (TLR1 to TLR10) were found to be expressed in porcine adrenal tissue under normal physiological conditions (Figure 3A). The TLR3 was the most abundantly expressed TLR, which was followed by TLR1 (Supplementary Table S2). The statistical analysis of TLR2, 4 mRNA expressions in adrenal gland revealed a main effect of LPS treatments (*P* < 0.01, Figures 3B,C). Treatment with our LPS preparations alone significantly increased levels of TLR2, 4 mRNA expression (*P* < 0.01 and *P* < 0.05, respectively). Transcriptional activation of TLR2 induced by the LPS was significantly relieved by ACTH pretreatment (*P* < 0.05). Furthermore, LPS alone significantly increased levels of GR mRNA expression (*P* < 0.05, Figure 3D). But no significant change was shown for LPS-induced GR or TLR4 mRNA expression by ACTH stimulation (Supplementary Table S1).

### Expressions of microRNA

All miRNAs that target TLR2 and TLR4 were predicted as shown in Table 3. The results showed that miR-338 and miR-146b had potential regulatory effects on TLR4. In contrast, highly reliable miRNAs that target TLR2 were not found. There were main effects of LPS (*P* < 0.01) and ACTH × LPS (*P* = 0.04) for ssc-miR-338 expression level by two-way ANOVA. Treatment with LPS alone extremely significantly decreased the ssc-miR-338 level (*P* < 0.01), yet the induction was not affected by ACTH pretreatment. In contrast, ACTH pretreatment combined with LPS promoted the expression of ssc-miR-146b significantly (*P* < 0.05) compared with the vehicle group despite no changes being detected with LPS treatment alone (Supplementary Table S1).

**Table 3:** Effects of LPS/ACTH on miRNA expressions targeting TLR4 gene in porcine adrenal tissue.

**Target gene**	**miRNA**	**ACTH -**		**ACTH** **+**		**S.E.M**.	***P*****–value**		
		**LPS -**	**LPS+**	**LPS -**	**LPS+**		**ACTH**	**LPS**	**ACTH × LPS**
TLR4	ssc-miR-338	1.00^a^	0.44^c^	0.71^b^	0.52^bc^	0.04	0.243	0.000	0.040
	ssc-miR-146b	1.00^b^	1.41^ab^	0.75^b^	2.45^a^	0.31	0.349	0.020	0.134

*Means with different letters (a, b, c) are significantly different (P <0.05) from each other, n = 5–6*.

*Different superscript letters indicate significant differences between the same column. Data are expressed as mean ± SEM*.

## Discussion

Lipopolysaccharide is known to stimulate an inflammatory response that induces production of cytokines. As expected, the body temperature was increased in all the LPS-treated pigs compared to the control group in our inflammation model of pig (27). And it was found in the present study that LPS upregulated expressions of IL-1β, IL-6, TNF-α, IL-10, and COX-2 mRNA in the adrenal gland of pigs. These results indicate that a strong inflammatory reaction may be induced locally in adrenal gland tissue. However, we found that, among all the cytokines examined, only levels of IL-1β, IL-10 mRNA were altered post LPS treatment in lung tissue (data not shown). LPS upregulated levels of pulmonary IL-1β and IL-10 gene mRNAs, but ACTH treatment has no significant effect on them. As for other genes related to inflammation, no significant differences were observed in the expression of IL-6, TNF-α, and COX-2 mRNAs post ACTH or LPS treatment. This suggests that the inflammatory response induced by LPS treatment is tissue-specific in our study. Similar results are reported in a previous publication that cytokine responses to LPS are age- and tissue-dependent in neuroendocrine tissues of neonatal pigs (33). Some previous studies on mice or rats have shown that ACTH attenuated LPS-induced systematic inflammatory response (34, 35). For example, it was reported that treatment with ACTH (1–24) sympathectomy attenuated LPS-induced increases of IL-1β, IL-6, and IL-10 levels in the plasma of rats. In accordance with this, we also found that ACTH pretreatment blocked intra-adrenal IL-6 protein secretion induced by LPS. Therefore, our finding provides *in vivo* evidence that the stress mimicked by continuous ACTH treatment may play a role in alleviating inflammatory reaction in the local adrenal gland.

The inflammatory response elicited by LPS mainly through activation of TLR4 has been well-documented in previous studies (36–39). It is also found that TLR2 can be upregulated by LPS stimulation in hemorrhagic shock mice (40), suggesting that both TLR2 and TLR4 can be activated by their LPS preparation. Previous studies have shown that expressions of TLR2 and TLR4 were increased by LPS treatment in the mouse adrenal gland, which further promotes the secretion of IL-1, IL-6, and TNF-α (12, 13). In accordance, we found that LPS activated mRNA expression of TLR2 and TLR4 in porcine adrenal gland tissue. However, it should also take into account the contamination of commercial LPS preparations (11). Indeed, LPS is the classical ligand of TLR4, and TLR2 should not be activated by pure LPS. Actually, commercial LPS preparation was always contaminated with TLR2 ligands, such as lipopeptides, that can activate TLR2 pathways (11). In the present work, we just used LPS to stimulate the inflammatory response rather than to specifically study the TLR4 pathway. Thus, commercial LPS was used in the present work. In addition, our findings provided *in vivo* evidence that ACTH pretreatment downregulated expressions of TLR2 induced by our LPS preparations in pig model, which was coupled with changes of IL-6 protein levels. These results may suggest that stress stimulated by continuous ACTH treatment prevented the transcriptional activation of TLR2 and, thus, protected the porcine adrenal gland against excessive inflammation.

Many studies have demonstrated that activated TLRs in organ injury are associated with oxidative stress (41, 42). NO (nitric oxide) is an important inflammatory mediator derived from NOS, including induced NOS (iNOS) and constitutive NOS (cNOS) (43). Actually, the regulation of NO production by cNOS and iNOS was a complex issue, and it was reported that the activity of cNOS was regulated by LPS and some cytokines in the mouse vascular endothelial cell line (44). It was also documented that the cNOS activity showed a 4.3-fold decrease post *H. pylori* LPS treatment in rat gastric mucosal cells while ghrelin countered the LPS-induced change (45). This result is consistent with our finding. Here, we found that LPS treatment downregulated cNOS levels, which was significantly relieved by ACTH pretreatment, thus resulting in an ACTH × LPS effect for cNOS level. These results indicate that the strong inflammatory reaction induced by LPS may decrease cNOS activity in local adrenal gland tissue although ACTH pretreatment alleviates the effect of LPS. NO is predominantly produced by iNOS in the late phase of inflammation (43, 46). Here, we found LPS treatment increased levels of iNOS protein secretion in the adrenal gland but decreased T-SOD activity without an ACTH × LPS effect. These results suggest that LPS may promote local iNOS activity and damage the activity of the antioxidant enzymes, which were consistent with the result of over-activation inflammation in local adrenal gland tissue. But ACTH has no effect on activity of iNOS and antioxidant enzymes.

Studies in mouse brains indicated that miR-146a and let-7b miRNAs were upregulated, but miR-338-3p miRNA expressions were downregulated in prion disease (17). It was also reported that miR-338-3p miRNA expression was downregulated during LPS-induced inflammation in mouse lung tissue (18). In accordance, here, we found that LPS injection alone extremely significantly decreased the ssc-miR-338 level in our pig model. However, it's puzzling that the LPS-induced inhibition of ssc-miR-338 was not affected by ACTH pretreatment by one-way ANOVA although with an ACTH × LPS effect. Here, we found that transcriptional activation of TLR4 induced by LPS was not affected by ACTH pretreatment. Thus, the result of ssc-miR-338 is consistent with our findings of TLR4 expression. It has been reported in several studies that the miR-146 family targeting various pro-inflammatory molecules in the TLR4 signaling pathway plays a negative regulatory role in inflammatory response (21, 22). In line with these findings, here, we found that ACTH combined with LPS stimulation upregulated miR-146b level. But inconsistent with expectation, transcriptional activation of TLR4 induced by LPS did not changed significantly by ACTH pretreatment. In fact, the miR-146 family might be the LPS primary response gene that targeted various pro-inflammatory molecules besides TLR4 (21). It was reported that 76 miRNA were significantly upregulated, and 35 miRNAs were downregulated at different time points post LPS treatment in mice, that targeted various pro-inflammatory molecules (18). Thus, here the ACTH-stimulated miR-146 or ssc-miR-338 regulatory circuit may fine-tune LPS-induced inflammatory signaling by targeting other inflammatory molecules.

The subcellular structure of adrenal mitochondria is closely related to the release of steroid hormones (11, 12). A defined spatial and conformational arrangement of mitochondria, which maintains normal electron transfer and cytochrome P450 activity, is required for the synthesis of glucocorticoid (11). Lipopolysaccharide impaired the mitochondrial structure in the pig adrenal gland in our study, which was restored to normal tubovesicular structure by ACTH pretreatment. The morphological alterations of mitochondria suggest mitochondrial dysfunctions post LPS treatment. The change of adrenal mitochondrial structure was consistent with cortisol levels in serum and adrenal gland tissue. This phenomenon may represent a compensatory mechanism for maintaining basal corticosterone release despite impaired adrenocortical function. The altered structure of the adrenals may reflect the enhanced synthesis and release of cortisol in the inflammatory state. We speculate that continuous ACTH treatment alleviates LPS-stimulated damage to mitochondria so that intra-adrenal cortisol can return to baseline levels.

The HPA axis is known to be activated by a variety of inflammatory insults, and innate immune-endocrine interactions and resultant GC-GR mediated action are critical for maintaining the homeostasis of immune response (33, 47). Bacterial invasion induces the secretion of cytokines, such as TNF-α, IL-1, and IL-6, which can stimulate the HPA axis to release corticotrophin-releasing hormone (CRH) and arginine vasopressin in the hypothalamus. Next, CRH stimulates the anterior pituitary to synthesize and release ACTH, which promotes the secretion of GC in the adrenal gland (48). GCs themselves exert a negative feedback regulation to suppress the HPA axis, which leads to a shift from pro-inflammatory immune responses to anti-inflammatory immune responses in circulation (49, 50). Actually, the HPA axis was stimulated directly by LPS that has been implicated in previous studies, including in the paraventricular nucleus of the hypothalamus (48), the anterior pituitary (51), or the adrenal glands (11). The activated HPA axis produced pro-inflammatory cytokines, such as TNF-α, NO, and IL-6, which involved in modulation of secretion of ACTH leading to downstream GC release. Consistent with this, here we found an exacerbated inflammatory response in local adrenal tissue and increased serum TNF-α concentration (data not shown) after LPS stimulation, accompanied by elevated cortisol levels at 6 h post LPS injection. Adrenal TLR2 and TLR4 should be tightly controlled in order to keep the balance between the adrenal endocrine and innate immune response, which are crucial for host survival (11, 12). Therefore, in the present study, activated TLR2 and TLR4 pathways by our LPS preparations may stimulate release of cytokines in local adrenal gland tissue and serum, and circulated cytokines may eventually promote cortisol release via stimulating HPA axis in a negative feedback loop.

Previous study has shown that an inflammatory transcription factor, namely NF-interleukin 6 (NF-IL-6), was activated in the anterior pituitary lobe not only by inflammatory LPS stimulation, but also by a novel environment stress that caused low-grade inflammatory responses in the brain and the anterior pituitary (51). However, in the present study, no adrenal response was observed by ACTH injection alone. This may be due to the fact that the consecutive exogenous ACTH treatment could reduce the adrenal gland sensitivity to ACTH stimulation. Interestingly, here, ACTH pretreatment relieved inflammation in adrenal gland upon exposure to our LPS preparations to a certain extent and, ultimately, restored systemic and local cortisol to basal levels at 6 h post LPS injection. Serum cortisol levels were increased at 2 h post LPS treatment and remained elevated at 6 h. However, pretreatment with ACTH significantly decreased the LPS-induced upregulation of cortisol and restored cortisol to the basal level at 6 h post LPS treatment (data not shown). It may suggest that the chronic stress stimulated by our consecutive ACTH treatment may keep the balance between the innate immune and adrenal endocrine response in the local adrenal gland post LPS injection. However, the adrenal response was in a time-dependent manner, and variations at various time points need to be investigated in a further study.

Previous studies have shown that transcriptional activation of TLR2 and TLR4 depend on CD14 and LPS-binding protein (LBP) (52). Signal transduction pathways, such as nuclear factor-kappa B (NF-κB) and mitogen-activated protein kinase (MAPK), were activated by TLR4 via a series of signal cascade reactions, which ultimately activate the inflammatory response (53). Classically, GC mediated-GR nuclear translocation plays a crucial role of anti-inflammation (54). Therefore, further investigations are needed to delineate profiles of TLR, GR protein, NF-κB, and MAPK signal transduction pathway molecules as well as the complex regulatory network of GR and TLR.

## Conclusions

Taken together, our results demonstrate that ACTH pretreatment seems to attenuate LPS-induced mitochondria damage and inflammation that decreased cNOS activity in the adrenal gland and ultimately returned local adrenal cortisol to basal levels at 6 h post LPS injection although ACTH had no effect on the LPS-induced iNOS secretion and inhibition of ssc-miR-338 and T-SOD activity. This suggests that moderate stress stimulated by repeated ACTH pretreatment may be beneficial for animals to resist inflammation in the adrenal gland. This study will deepen the knowledge about the relationship between inflammation and stress and provide a theoretical basis for the control of porcine inflammation and the study of animal welfare.

## Data Availability Statement

The datasets generated for this study are available on request to the corresponding author.

## Ethics Statement

All animal procedures were approved by the Institutional Animal Care and Use Committee (IACUC) of Nanjing Agricultural University. The protocol of this study was reviewed and approved specifically, with the project number 2012CB124704. The slaughter and sampling procedures strictly followed the Guidelines on Ethical Treatment of Experimental Animals (2006) No. 398 set by the Ministry of Science and Technology, China and the Regulation regarding the Management and Treatment of Experimental Animals (2008) No. 45 set by the Jiangsu Provincial People's Government.

## Author Contributions

ZS and XZ designed this study, guided the experiment, and analyzed data. ZS, DC, and XY have been involved in the whole experiment process and drafting and revising the manuscript. YS, CY, HZ, and XL participated all the experiments and performed the statistical analysis. QS, YJ, and YX helped for the sampling process and made contributions to acquisition of data. All authors contributed to manuscript revision, read and approved the submitted version.

## Conflict of Interest

The authors declare that the research was conducted in the absence of any commercial or financial relationships that could be construed as a potential conflict of interest.

## Abbreviations

TLR: toll-like receptor
GAPDH: glyceraldehyde-3-phosphate dehydrogenase
GR: glucocorticoid receptor
IL: interleukin
TNF-α: tumor necrosis factor-α
COX-2: cyclooxygenase-2
T-NOS: total superoxide dismutase
iNOS: inducible nitric oxide synthase
cNOS: constitutive nitric oxide synthase
T-SOD: total superoxide dismutase
XOD: xanthineoxidase
CAT: catalase
LPS: lipopolysaccharide
miRNA: microRNA.
