# Supplementary material for: Stress Response Simulated by Continuous Injection of ACTH Attenuates Lipopolysaccharide-Induced Inflammation in Porcine Adrenal Gland
Source: Front Vet Sci. 2020 Jun 26;7:315. doi: 10.3389/fvets.2020.00315 (PMC7333078; doi:10.3389/fvets.2020.00315)
Supplement: Supplementary file 1 [file Data_Sheet_1.PDF]

Supplementary Material

Supplementary Material S1: Total RNA extraction and Real-time quantitative PCR (QPCR) reactions

Total RNA was extracted from adrenal gland tissues using Trizol reagent (Invitrogen, Carlsbad, CA) according to our previous description. The total tissue RNA was collected by using TRIzol kit. In order to ensure OD260/OD280 range from 1.8 to 2.0, spectrophotometer (Nano Drop, ND-1000) was used to determine the total RNA concentration and purity. In order to ensure that the extracted RNA did not degrade, 1.4% agarose-formaldehyde denaturation gel electrophoresis was used. The obtained bands must be clear without tailing phenomenon, and the ratio of 28S to 18S band gray scale was 2:1.

From each sample 1 μg of total RNA was converted to cDNA using PrimeScript® RT reagent kit with gDNA Eraser (Takara, Dalian, China). This kit includes a step for the elimination of genomic DNA. The primers used are listed in Table 1. Also, QPCR reactions and gene expression levels of mRNA were performed using the SYBR Green QPCR Master Mix (TOYOBO Ltd., Japan) by using a Mx3000P QPCR system (Stratagene, USA) according to the instruction manual. A subsequent step to generate a dissociation curve was used to verify that a single amplicon was generated. The reaction procedures are as follows:

Segment 1：predenaturation	Segment 2：PCR reactions	Segment 3：
95℃ 30 s	95℃ 5 s	Dissociation Curve
1 Cycle	60℃ 20 s	
	40 Cycles	

Glyceraldehyde-3-phosphate dehydrogenase (GAPDH) was chosen as a reference gene for normalization. All the mRNA expression levels were presented as the fold change relative to the average values in the CC group.The CT value was analyzed by $2^{-\Delta\Delta CT}$ method with formula as follows: ΔΔCT=（CT.Target− CT.GAPDH）X −（CT.Target −CT.GAPDH）control. X represents any sample.

Supplementary Table S1: Qualitative and quantitative analysis of stress and inflammation response Indexes in local porcine adrenal tissue post lipopolysaccharide/ACTH treatment

Items	Vehicle			LPS			ACTH			ACTH+LPS		
	Mean	SEM	CV	Mean	SEM	CV	Mean	SEM	CV	Mean	SEM	CV
T-NOS (U/mg protein)	1.70	0.212	0.305	1.12	0.079	0.173	1.38	0.113	0.183	1.42	0.145	0.227
iNOS (U/mg protein)	0.36	0.058	0.393	0.43	0.061	0.345	0.31	0.039	0.286	0.47	0.060	0.285
cNOS (U/mg protein)	1.34	0.162	0.295	0.69	0.041	0.148	1.07	0.080	0.166	0.95	0.089	0.210
T-SOD (U/mg protein)	87.94	5.886	0.164	57.94	6.573	0.278	76.28	2.931	0.086	55.08	5.583	0.227
XOD (U/g protein)	35.58	3.179	0.219	28.63	1.934	0.165	32.91	1.887	0.128	32.02	3.980	0.278
CAT (U/mg protein)	0.90	0.066	0.180	0.88	0.067	0.187	0.80	0.032	0.089	0.90	0.059	0.146

ssc-miR-338	1.03	0.127	0.276	0.45	0.033	0.181	0.73	0.088	0.298	0.54	0.096	0.401
ssc-miR-146b	1.52	0.517	0.761	2.14	0.722	0.828	1.14	0.310	0.665	3.72	0.861	0.517
IL-1β mRNA	1.09	0.246	0.551	1.77	0.052	0.071	0.85	0.077	0.222	2.18	0.316	0.324
IL-6 mRNA	1.04	0.132	0.310	8.02	1.415	0.432	1.10	0.207	0.460	4.97	1.020	0.459
TNF-α mRNA	1.01	0.057	0.139	1.62	0.203	0.307	0.84	0.120	0.348	1.30	0.234	0.403
IL-10 mRNA	1.04	0.133	0.312	8.06	2.042	0.621	0.79	0.116	0.360	6.12	2.109	0.770
COX-2 mRNA	1.06	0.160	0.369	1.74	0.267	0.376	0.79	0.145	0.449	1.54	0.234	0.340
IL-6 protein (pg/mg protein)	67.1	5.45	0.199	109.2	30.21	0.677	56.1	3.41	0.136	54.2	5.80	0.239
Cortisol (ng/mg protein	114.6	16.75	0.358	188.4	66.49	0.864	95.2	8.72	0.205	76.7	6.32	0.184
TLR2 mRNA	1.06	0.144	0.334	4.38	0.629	0.352	0.69	0.053	0.187	3.06	0.298	0.218
TLR4 mRNA	1.04	0.128	0.301	5.64	0.972	0.422	0.85	0.054	0.156	3.87	0.737	0.425

GR mRNA	1.01	0.066	0.160	1.74	0.227	0.319	1.08	0.123	0.279	1.52	0.120	0.176

TLR = toll-like receptor; GR = glucocorticoid receptor; IL = interleukin; TNF-α = tumor necrosis factor-α; COX-2 = cyclooxygenase-2; T-NOS = total superoxide dismutase; iNOS = inducible nitric oxide synthase; cNOS = constitutive nitric oxide synthase; T-SOD = total superoxide dismutase; XOD = xanthineoxidase; CAT = catalase.

Supplementary Table S2: Qualitative and quantitative analysis of TLR1-10 mRNA levels in local porcine adrenal tissue under normal physiological condition

mRNA levels	Mean	SEM	CV
TLR1	1.02	0.087	0.210
TLR2	0.77	0.072	0.230
TLR3	1.31	0.181	0.337
TLR4	0.46	0.046	0.241
TLR5	0.84	0.019	0.055
TLR6	0.14	0.010	0.174
TLR7	0.17	0.015	0.210
TLR8	0.28	0.031	0.266
TLR9	0.05	0.004	0.232
TLR10	0.11	0.007	0.147
